# Supplementary material for: WordCommentsAnalyzer: A windows software tool for qualitative research
Source: F1000Res. 2018 Sep 4;7:536. Originally published 2018 May 3. [Version 2] doi: 10.12688/f1000research.14819.2 (PMC6137411; doi:10.12688/f1000research.14819.2)
Supplement: Supplementary file 3 [file f1000research-7-17614-s0002.tgz › b2a79496-4ada-47c4-9682-a99ce867836d.zip!/Eight Iranian Health Journals/Archives of Rehabilitation_46.docx]

© Archives of Rehabilitation. This work is licensed under CC BY-NC 4.0 (https://creativecommons.org/licenses/by-nc/4.0/).

Volume & Issue: دوره 9 - شماره 1

Title: تأثیر سه روش درمانی بوبت، آموزش نمونشی و آموزش به والدین بر فعالیت‌های روزمره زندگی کودکان فلج مغزی

http://rehabilitationj.uswr.ac.ir/article-1-221-fa.html

هدف: این مطالعه به منظور مقایسه تأثیر سه رویکرد درمانی بوبت، آموزش نمونشی و آموزش به والدین بر فعالیتهای روزمره زندگی کودکان فلج مغزی 5-3 ساله آموزش‌پذیر انجام گرفت. روش بررسی: در این مطالعه شبه تجربی و مداخله‌ای که به صورت قبل و بعد انجام شد، جامعه هـدف کودکان فلج مغزی 5-3 سال شهر تهران و نمونه‌های در دسترس کودکان مراجعه کننده به کلینیک‌های بنیاد توانبخشی ولعیصر(عج) بودند که از بین آنها 45 کودک که شرایط ورود به مطالعه را داشتند، با در نظر گرفتن شرایط همگنی (سن، جنس، هوشبهر و نوع اختلال) به‌صورت ساده و هدفمند انتخاب و به روش تصادفی ساده به سه گروه 15 نفره تخصیص یافته و مداخلات درمانی بوبت، آموزش نمونشی و آموزش به والدین بر روی آنها صورت گرفت. ابزارهای مورد استفاده شامل آزمونهای گودیناف جهت تعیین میزان هوشبهر و آمـوزش‌پـذیر بودن کودکان و گـزارش ارزیابی پیشرفـت مـددجو (CDER) جهت ارزیابی عملکرد فعالیت‌های روزمره زندگی (ADL) بودند. تجزیه وتحلیل آماری با استفاده از آزمونهای آماری ویلکاکسون، کروسکال – والیس و من- ویتنی انجام شد. یافته‌ها: هر سه گروه پیشرفت معنی‌داری در انجام فعالیتهای روزمره زندگی بعد از انجام مداخلات نسبت به قبل از آن داشتند (P<0/001). تفاوت معنی‌داری بین سه گروه از لحاظ عملکرد کل ADL وجود داشته (P<0/001) و روشهای درمانی به ترتیب اثرگذاری عبارت بودند از: آموزش نمونشی، آموزش به والدین و بوبت. رابطه معنی‌داری بین اختلاف عملکرد سه گروه، در 13 خرده مهارت از 17خرده مهارت فعالیتهای روزمره زندگی وجود داشت (p<0/05). نتیجه‌گیری: رویکردهای بوبت، آموزش نمونشی و همچنین آموزش به والدین موجب پیشرفت مهارتهای فعالیتهای روزمره زندگی می‌شوند، اما در رویکرد درمانی آموزش نمونشی، تأکید برآموزشهای گروهی به مددجویان، سبب بهبود بیشتر تعاملات اجتماعی و ارتباطی و همچنین وجود برنامه و آموزشهای عملی، باعث پیشرفت آنها در انجام مهارتهای روزمره زندگی می‌گردد.

Title: مقایسه شاخص‌های تنفس گفتاری در مجروحین شیمیایی ناشی از گاز خردل با افراد عادی

http://rehabilitationj.uswr.ac.ir/article-1-222-fa.html

هدف: نقش تنفس فقط به عملکرد حیاتی آن محدود نمی‌شود، بلکه از نظر گفتاری نیز اهمیت به سزایی دارد. هدف این مطالعه بررسی شاخصهای تنفس گفتاری در مجروحین شیمیایی ناشی از گاز خردل و مقایسه آن با افراد عادی می‌باشد. روش بررسی: این پژوهش یک مطالعه مقطعی- مقایسه‌ای ازنوع مورد- شاهدی می‌باشد. گروه مورد که به روش نمونه‌گیری در دسترس انتخاب شد، شامل 19نفر مرد مجروح شیمیایی ناشی از گاز خردل مراجعه‌کننده به بیمارستان بقیه‌الله(عج) تهران بود و گروه شاهد شامل 20 نفر مرد سالم از کارکنان دانشگاه علوم بهزیستی و توانبخشی، که از طریق همتاسازی با گروه مورد انتخاب شد. ابزار مورد استفاده برای اندازه‌گیری شاخصهای تنفس گفتاری دستگاه اس.تی.یک دیسفونیا ساخته شده توسط آزمایشگاه دستگاههای G.M بود که بوسیله آن متغیرهای ظرفیت حیاتی، زمان آواسازی، حجم آواسازی، متوسط سرعت جریان هوا درطول آواسازی وحجم کلی بازدم اندازه‌گیری شد. همچنین مقادیر شاخص سرعت آوایی و میزان آواسازی نیز مورد سنجش قرار گرفت. داده‌های حاصل از تحقیق بوسیله آزمون آماری تی - مستقل تجزیه وتحلیل شد. یافته‌ها: اختلاف آماری معناداری بین گروه مورد وشاهد در متغیرهای ظرفیت حیاتی (P<0/001)، زمان آواسازی (P<0/001)، حجم آواسازی (P<0/001)، شاخص سرعت آوایی (P=0/004)، حجم کلی بازدم (P<0/001) و میزان آواسازی (P=0/046) وجود داشت، ولی در متوسط سرعت جریان هوا بین دو گروه اختلاف معنی‌‌داری وجود نداشت (P=0/615). نتیجه‌گیری: گازهای شیمیایی علاوه بر تأثیر بر تنفس حیاتی، بر عملکرد تنفس گفتاری نیز مؤثر می‌باشند. این امر می‌تواند نقش مهمی را در معیارسنجی و تعیین شاخصهای مختل شده آوایی ایفا نماید، به‌نحوی که گفتار درمانگر می‌تواند خدمات گفتاری دقیق‌تری را به این بیماران ارائه کند.

Title: بررسی تأثیر برنامه‌های بازتوانی قلبی کوتاه‌مدت بر میزان عود بیماری، مراجعه و بستری مجدد در مبتلایان به انفارکتوس میوکارد

http://rehabilitationj.uswr.ac.ir/article-1-223-fa.html

هدف: انفارکتوس میوکارد از شایع‌ترین علل مرگ و میر و ناخوشی در جهان امروز است که معلولیت‌ها و زیان‌های اقتصادی و اجتماعی فراوانی به همراه دارد. یکی از روشهای پیشگیری ثانویه، انجام برنامه‌های بازتوانی قلبی با مشارکت پرستاران می‌باشد. هدف این پژوهش تعیین تأثیر برنامه‌های کوتاه‌مدت بازتوانی قلبی بر میزان عود بیماری، مراجعه و بستری مجدد بیماران انفارکتوس میوکارد است. روش بررسی: پژوهش حاضر از نوع نیمه‌تجربی و نمونه‌های پژوهش 66 بیمار مبتلا به انفارکتوس میوکارد، بستری در مراکز درمانی آموزشی شهر اهواز می‌باشند که با روش نمونه‌گیری مبتنی برهدف انتخاب و به طور تصادفی ساده به دو گروه مداخله (32نفر) و شاهد (34نفر) تقسیم شدند. اطلاعات دموگرافیک به وسیله چک‌لیست از هردوگروه جمع‌آوری شد. افراد گروه مداخله، تحت برنامه توانبخشی و بازتوانی قلبی به مدت 8 هفته قرار گرفتند. پس از 8 هفته اطلاعات مربوط به میزان وقوع بستری مجدد و عود بیماری در طی این مدت در بیماران هردو گروه به وسیله چک‌لیست جمع‌آوری و با استفاده از آزمونهای آماری کای اسکوئر و من ویتنی مورد تجزیه و تحلیل قرارگرفت. یافته‌ها‌:‌ میزان عود بیماری در بیماران انفارکتوس میوکارد در دو گروه مداخله و شاهد و نیز تعداد موارد بستری مجدد در دو گروه تفاوت معنی‌دار داشت (P<0/001). نتیجه‌گیری: شرکت در برنامه‌های توانبخشی و بازتوانی قلبی، تعداد موارد عود بیماری، مراجعه و بستری مجدد در مبتلایان به انفارکتوس میوکارد را کاهش می‌دهد.

Title: تأثیر موسیقی و حرکات موزون بر دامنه توجه دانش‌آموزان دختر کم‌توان ذهنی

http://rehabilitationj.uswr.ac.ir/article-1-224-fa.html

هدف: این پژوهش به منظور بررسی تأثیر موسیقی و حرکات موزون بر دامنه توجه دانش‌آموزان دختر کم‌توان ذهنی صورت گرفت. روش بررسی: در این پژوهش که از نوع مطالعات تجربی- مداخله‌ای بوده و به صورت پیش‌آزمون- پس‌آزمون با گروه کنترل انجام شد، 26 دانش‌آموز دختر کم‌توان ذهنی از پایه‌های چهارم و پنجم مرکز آموزش استثنایی دانش شهر ری (سال تحصیلی 86-1385) با استفاده از روش نمونه‌گیری در دسترس انتخاب و به صورت تصادفی تعادلی به دو گروه مساوی تخصیص یافتند و سپس با انتساب تصادفی، مداخله فعالیت‌های موسیقایی (شامل موسیقی و اجرای حرکات موزون) در گروه آزمون به مدت 15 هفته و هر هفته دو جلسه اجرا گردید. در این مدت گروه کنترل تحت این تمرینات قرار نداشته و فقط برنامه درسی معمولی را دریافت نمودند. برای هر دو گروه قبل و بعد از اجرای مداخلات، خرده‌آزمون تیک زنی از آزمون کتلر- لارنت – تیریو (KLT) به منظور سنجش دامنه توجه اجرا شد. به دلیل کم بودن تعداد آزمودنی‌ها در هر گروه، از آزمون‌های ناپارامتری یعنی از آزمون رتبه‌ای علامت دار ویلکاکسون برای مقایسه تفاوت میانگین‌ها در یک گروه و آزمون یو من‌ویتنی برای مقایسه میانگین‌ها در دو گروه استفاده شد. یافته‌ها: بین میانگین نمرات پیش‌آزمون و پس‌آزمون توجه در گروه آزمون، تفاوت معنی‌دار وجود داشت(P<0/001)، ولی در گروه کنترل این تفاوت، معنی‌دار نبود (P=0/367). همچنین میانگین نمرات توجه دو گروه کنترل و آزمون پس از مداخله تفاوت معنی‌دار داشته و عملکرد گروه آزمون بهتر بود (P=0/002). در حالی که بین میانگین نمرات دو گروه قبل از مداخله تفاوت معنی‌داری وجود نداشت (P=0/520). نتیجه‌گیری: با استناد به داده‌های به‌دست آمده می‌توان نتیجه گرفت، با اجرای فعالیت‌های موسیقایی، دامنه توجه دانش‌آموزان کم‌توان ذهنی بهبود می‌یابد. البته لازم به توضیح است که با توجه به نوع نمونه‌گیری، امکان تعمیم نتایج این تحقیق کاهش می‌یابد.

Title: میزان استفاده از وسایل کمک‌بینایی در افراد کم‌بینا پس از تجویز در دو کلینیک صبا و خزانه

http://rehabilitationj.uswr.ac.ir/article-1-225-fa.html

هدف: اگرچه تحقیقات بسیاری نشان داده که وسایل کمک‌بینایی بر افزایش دید افراد کم‌بینا مؤثر است، ولی برخی از افراد علیرغم این تأثیر مثبت به دلایل مختلف از این وسایل استفاده نمی‌کنند. لذا این پژوهش با هدف بررسی میزان استفاده از وسایل کمک‌بینایی در زندگی روزمره این افراد انجام شد. روش بررسی: در این پژوهش توصیفی که در دو کلینیک صبا و خرانه (وابسته به دانشگاه علوم بهزیستی و توانبخشی و بهزیستی استان تهران) انجام شد، تعداد 50 نفر از افراد کم‌بینا از بین مراجعه کنندگان این دو مرکز طی سالهای 84 و 85 ، که واجد شرایط لازم بودند، به صورت در دسترس انتخاب شدند. پس از انجام معاینات بینایی سنجی، پرسشنامه اول که در آن سئوالاتی راجع به وضعیت انجام کارهای روزمره وابسته به بینایی قرارداشت، با پرسش از فرد تکمیل ‌شده و سپس وسیله مورد نیاز تجویز و از وی خواسته می‌شد تا بعد از حداقل سه ماه مجدداً مراجعه کند که در این مرتبه پرسشنامه دوم که حاوی سئوالاتی مبنی بر تغییر وضعیت کارهای چشمی با توجه به استفاده از وسیله مذکور بود از وی پرسیده می‌شد. مقایسه پاسخهای فرد به این دو پرسشنامه، مبنای میزان استفاده از وسیله با توجه به تأثیر آن در کارهای چشمی بود. داده‌های حاصل با استفاده از آزمونهای آماری ضریب همبستگی اسپیرمن، کای اسکوئر و ویلکاکسون مورد تحلیل قرار گرفت. یافته‌ها: ضریب همبستگی اسپیرمن بین میزان استفاده از وسایل کمک‌بینایی نزدیک‌بینی «مطالعه» و مدت و دفعات انجام مطالعه به ترتیب 0/491 و 0/520 با مقادیر احتمال P=0/003 و P=0/002 و نشان از وجود ارتباط معنی‌دار و افزایشی بود. تفاوت معنی‌داری بین میانگین میزان تردد و شناسایی محیط و اشیا «دوربینی»‌ در قبل و بعد از استفاده از وسیله کمک‌بینایی دور وجود داشت (P=0/002). همچنین ضریب همبستگی اسپیرمن بین میزان استفاده از وسیله کمک‌بینایی و میزان تردد و شناسایی 0/499 و بیانگر رابطه مثبت بود. بین میزان استفاده از وسیله کمک‌بینایی پس از تجویز و تحصیلات افراد نیز رابطه معنی‌داری به‌دست آمد. 54 درصد از گیرندگان وسایل کمک بینایی از به کاربرد آن در انظار دیگران اجتناب می‌کردند (P=0/011). نتیجه‌گیری: علیرغم تأثیر مسلم وسایل کمک‌بینایی در افزایش بینایی افراد کم‌بینا و کیفیت زندگی آنان، دلایل مختلف، تعداد قابل توجهی از این افراد از استفاده از آنها طفره رفته و یا کمتر استفاده می‌کنند.

Title: بررسی تأثیر حرکات اصلاحی بر اتساع قفسه سینه در دختران کیفوتیک 15-11 ساله

http://rehabilitationj.uswr.ac.ir/article-1-226-fa.html

هدف: این پژوهش با هدف تعیین تأثیر یک دوره حرکات اصلاحی برروی اتساع قفسه سینه دختران کیفوتیک 15 – 11 ساله انجام گرفت. روش بررسی: مطالعه شبه تجربی حاضر یک کارآزمایی بالینی است که بر روی 50 نفر از دختران کیفوتیک 15-11 ساله که به روش نمونه‌گیری ساده از جامعه در دسترس انتخاب شده و به صورت تصادفی ساده به دو گروه 25 نفری آزمایش وکنترل تقسیم شده بودند، انجام شد. روی گروه آزمایش یک دوره حرکات اصلاحی اعمال شد، ولی روی گروه کنترل این حرکات اصلاحی اعمال نگردید و اتساع قفسه سینه قبل و بعد از حرکات اصلاحی در دو گروه مورد اندازه‌گیری قرارگرفت. داده‌های حاصل با استفاده از آزمونهای آماری کای اسکوئر، تی زوجی و تی مستقل مورد تجزیه و تحلیل قرار گرفت. یافته‌ها‌: اختلاف معنی‌داری در میزان اتساع قفسه سینه قبل و بعد از حرکات اصلاحی در گروه آزمایش وجود داشت (P<0/001)، ولی در گروه کنترل اختلاف معنی‌داری مشاهده نگردید (P=0/746). هم‌چنین بین دو گروه نیز بعد از اعمال مداخله اختلاف معنی‌داری درمیزان اتساع قفسه سینه دیده شد (P=0/02). نتیجه‌گیری: انجام حرکات اصلاحی موجب بهبود اتساع قفسه سینه دختران کیفوتیک می‌شود. اگرچه به دلیل نوع نمونه‌گیری و محدودیت تعمیم‌پذیری نتایج، نیاز به تکرار این گونه تحقیقات وجود دارد.

Title: تأثیر بی‌‏حسی انتخابی موقتی همراه با بازآموزی حسی روی نتایج آستانه حس لمس سبک/فشار، بعد از ترمیم اعصاب مدیان/اولنار

http://rehabilitationj.uswr.ac.ir/article-1-227-fa.html

هدف: نتایج حسی، پس از ترمیم عصب در بزرگسالان، عموماً ضعیف می باشد. مطابق با نتایج به‌دست آمده قبلی و طرح این فرضیه که آوران زدایی ساعد نتایج حسی را ازطریق افزایش نمایش کورتیکال دست بهبود می بخشد، این مطالعه با هدف بررسی تأثیر بی حسی موضعی ساعد در ترکیب با انجام برنامه بازآموزی حسی بر روی نتایج حسی ضایعات اعصاب مدیان یا اولنار انجام شد. روش بررسی: این مطالعه به روش تجربی و از نوع کارآزمایی بالینی تصادفی دوسو کور می‌باشد. به روش احتمالی 13 بیمار که تحت جراحی ترمیمی اعصاب دست قرار گرفته بودند انتخاب و به روش تصادفی به دو گروه آزمون (6 نفر) و کنترل (7 نفر) تقسیم شدند. طی یک دوره دو هفته ای یک کرم بی حسی موضعی (لیدوکایین P) در گروه مورد و یک دارونما در گروه کنترل 4 بار بر روی وجه فلکسور ساعد دست آسیب‌دیده مورد استفاده قرار گرفت. برنامه بازآموزی حسی برای هر دو گروه یک ساعت پس از استعمال پماد و به مدت یک‌ساعت انجام شد. ارزیابی حسی در فواصل منظم، قبل از مداخله و پس از آخرین مداخله (پایان جلسه چهارم) در هر دو گروه انجام شد. برای داده های با توزیع غیر نرمال، از آزمون های ناپارامتری من ویتنی- یو و ویلکاکسون، به ترتیب برای مقایسه نتایج قبل و بعد درمان بین گروهی و درون گروهی استفاده گردید. یافته‌ها: گروه آزمون بهبودی قابل توجهی در مقایسه با گروه کنترل از نظر درک لمس/ فشار ثابت پس از دوره درمان، کسب نموده و اختلاف آنها معنی‌دار بود (P=0/03). نتیجه‌گیری: بی حسی موضعی ناحیه ساعد آسیب دیده در ترکیب با بازآموزی حسی می تواند نتایج حسی را پس از ترمیم عصب ارتقا دهد.

Title: شیوع لکنت در دانش‌آموزان دوزبانه مقاطع مختلف شهر جوان‌رود

http://rehabilitationj.uswr.ac.ir/article-1-228-fa.html

هدف: به منظور بررسی نقش عوامل زبان‌شناختی در ابتلا به لکنت و پاسخ‌گویی به نیازهای درمانی آن، برآورد میزان شیوع این اختلال در دوزبانه‌های ایرانی ضروری است. لذا هدف از مطالعه حاضر برآورد شیوع لکنت در دانش‌آموزان مقاطع مختلف شهر دوزبانه جوانرود به تفکیک جنس و مقطع تحصیلی است. روش بررسی: این مطالعه مقطعی از نوع توصیفی- تحلیلی است. تمام دانش‌آموزان مدارس عادی سه مقطع تحصیلی شهر جوانرود به صورت تمام شمار بررسی شدند. غربالگری اولیه جهت شناسایی دانش‌آموزان مبتلا به انواع اختلالات گفتاری توسط معلمان و با نظارت کارشناس گفتاردرمانی انجام گرفت و اطلاعات لازم در این زمینه در جلسات توجیهی به معلمین داده شد. از بین‌دانش‌آموزان معرفی شده توسط معلمان، موارد مبتلا به لکنت از طریق ارزیابی گفتار خودبه‌خودی، توصیف تصاویر داستانی و خواندن متن به دو زبان کردی و فارسی توسط کارشناس گفتاردرمانی بررسی و بر اساس علائم آسیب‌شناختی لکنت تشخیص داده شدند. داده‌های حاصل با استفاده از آزمونهای آماری کای اسکوئر و رگرسیون لجستیک مورد تحلیل قرار گرفت. یافته‌ها: از تعداد کل 11425 دانش‌آموز، 129 نفر مبتلا به لکنت بودند. این تعداد معادل 1/13 درصد کل جامعه مورد مطالعه است. شیوع لکنت در مقاطع ابتدایی، راهنمایی و دبیرستان به ترتیب 2/06، 0/87 و 0/5 درصد بود. شیوع لکنت بین پسران 1/13 درصد و بین دختران 0/88 درصد به‌دست آمد. نسبت ابتلای پسران به دختران 1/5 به 1 بود. شیوع لکنت بین مقاطع تحصیلی ابتدایی، راهنمایی و دبیرستان باهم تفاوت معنی‌دار داشت (P<0/001). تفاوت شیوع لکنت بین پسران و دختران از لحاظ آماری معنادار بود (P=0/034). نتیجه‌گیری: شیوع لکنت در بین دانش‌آموزان دوزبانه شهر جوانرود بیشتر از میزان گزارش شده به عنوان میانگین شیوع لکنت در جوامع یک زبانه (1 درصد) است. خطر ابتلا به لکنت در پسران بیشتر از دختران است.

Title: تأثیر فیدبک بینایی بر تعادل بیماران مبتلا به پارکینسون ایدیوپاتیک

http://rehabilitationj.uswr.ac.ir/article-1-229-fa.html

هدف: پارکینسون ایدیوپاتیک یک بیماری تخریبی و مزمن سیستم عصبی است که عدم تعادل بدن مشخصه اصلی آن می‌باشد. هدف این تحقیق بررسی تأثیر فیدبک بینایی بر تعادل بیماران پارکینسونی می‌باشد. روش بررسی: در این مطالعه مقطعی مقایسه‌ای که از نوع قبل و بعد می‌باشد، 16 بیمار پارکینسونی از نمونه‌های در دسترس به‌صورت تصادفی انتخاب و آزمونهای تعادلی با استفاده از دستگاه تعادلی بایودکس در دو حالت چشم باز و چشم بسته از آنان به‌‌عمل آمد. داده‌های حاصل با استفاده از آزمون آماری تی زوجی مورد بررسی قرار گرفت. یافته‌ها: بین حالتهای چشم باز و چشم بسته بیماران پارکینسون، اختلاف معنی‌داری در شاخصهای ثبات کلی (P=0/004)، ثبات قدامی – خلقی (P=0/009)، ثبات داخلی – خارجی (P=0/003) و مدت‌ زمان قرارگیری فرد در مناطق یک (P=0/001)، دو (P=0/003) و سه (P=0/001) از صفحه نیروی دستگاه تعادلی بایودکس وجود داشت، ولی مدت زمان قرارگیری در منطقه چهار (P=0/286) و بخش‌های اول (P=0/664)، دوم (P=0/075)، سوم (P=0/135) و چهارم (P=0/287) صفحه نیرو، در دو حالت مذکور اختلاف معنی‌‌داری در بیماران نداشت. نتیجه‌گیری: فیدبک بینایی باعث بهبود تعادل در حالت چشم باز نسبت به حالت چشم بسته در بیماران مبتلا به پارکینسون می‌شود و این بهبودی در شاخص‌های ثبات کلی، ثبات قدامی- خلفی و ثبات داخلی- خارجی کاملاً مشهود است.

Title: بررسی اختلالات شخصیت و ویژگی‌های شخصیتی مردان مبتلا به اختلال هویت جنسی

http://rehabilitationj.uswr.ac.ir/article-1-230-fa.html

هدف: هدف از این مطالعه بررسی اختلالات شخصیت و ویژگی‌های شخصیتی مردان مبتلا به اختلال هویت جنسی می‌باشد. شناسایی اختلالات شخصیت می‌تواند در بالا بردن کیفیت کمک به بیماران مبتلا مفید واقع شود. روش بررسی: این پژوهش تحلیلی – مقطعی به روش مقایسه‌ای و از نوع مورد – شاهدی و با نمونه‌گیری ساده و در دسترس می‌باشد. آزمودنی‌ها تعداد 40 نفر از مردان مبتلا به اختلال هویت جنسی بودند که به مرکز بهزیستی نواب صفوی تهران مراجعه می‌کردند. هم‌چنین 40 نفر از کارمندان مرد دانشگاه آزاد اسلامی واحد علوم و تحقیقات تهران که هیچ یک از ملاک‌های تشخیصی اختلال هویت جنسی را براساس راهنمای تشخیصی و آماری اختلالات روانی – ویرایش چهارم نداشتند، با توجه به همتاسازی با بیماران به عنوان گروه گواه انتخاب شدند. اختلالات شخصیت و فراوانی آنها با استفاده از پرسشنامه بالینی چند محوری میلون (MCMI-II) مورد بررسی قرار گرفت. داده‌ها با استفاده از آزمون مجذور کای و تی برای گروه‌های مستقل مورد تجزیه و تحلیل قرار گرفت. یافته‌ها: نتایج حاصل نشان داد که بیماران مبتلا به اختلال هویت جنسی در مقیاس‌های وابسته (P=0/038)، نمایشی (P<0/001)، ضداجتماعی (P=0/017)، منفعل-مهاجم (P=0/007)، مرزی (P<0/001) و پارانوئید (P=0/021) نمرات بالاتری را نسبت به گروه گواه به‌دست آورده و اختلاف آنها از لحاظ آماری معنی‌داری می‌باشد. نتیجه‌گیری: مطابق با یافته‌های حاصل، بیماران مبتلا به اختلال هویت جنسی، برخی ویژگیها و خرده مقیاسهای اختلالات شخصیتی را بیشتر از افراد عادی دارا بوده و درجاتی از اختلالات شخصیت در برخی از آنها بارز است.
